# Live imaging analysis of the growth plate in a murine long bone explanted culture system

**DOI:** 10.1038/s41598-018-28742-x

**Published:** 2018-07-09

**Authors:** Keisho Hirota, Akihiro Yasoda, Yugo Kanai, Yohei Ueda, Ichiro Yamauchi, Takafumi Yamashita, Yoriko Sakane, Toshihito Fujii, Nobuya Inagaki

**Affiliations:** Department of Diabetes, Endocrinology and Nutrition, Kyoto University Graduate School of Medicine, Kyoto, 606-8507 Japan

## Abstract

Skeletal growth in mammals, which owes the growth of an individual, occurs at the growth plate and to observe and analyze its dynamic growth is of high interest. Here we performed live imaging analysis of the growth plate of a fetal murine long bone organ culture using two-photon excitation microscopy. We could observe a dynamic growth of the growth plate of explanted fetal murine ulna, as well as the resultant linear elongation of the explants. As for the factors contributing to the elongation of the growth plate, the displacement length of each chondrocyte was larger in the prehypertrophic or hypertrophic zone than in the proliferative zone. The segmented area and its extracellular component were increased in both the proliferative and prehypertrophic-hypertrophic zones, whereas an increase in cellular components was only seen in the prehypertrophic-hypertrophic zone. C-type natriuretic peptide, a known positive stimulator of endochondral bone growth mainly targeting prehypertrophic-hypertrophic zone, augmented all of the factors affecting growth plate elongation, whereas it had little effect on the proliferation of chondrocytes. Collectively, the axial trajectory of each chondrocyte mainly owes cellular or extracellular expansion especially in prehypertrophic-hypertrophic zone and results in growth plate elongation, which might finally result in endochondral bone elongation.

## Introduction

The skeleton is crucial for retaining the shape of an individual organism and several modes of skeletogenesis have developed in the process of evolution^1^. Mammals have adopted a process called endochondral bone formation, in which a cartilaginous anlage is formed first and then replaced by calcified bone tissue. Endochondral bone formation is a precisely controlled dynamic process through which bones are formed and then elongate longitudinally^2^. In this process, chondrocytes in the cartilaginous anlage undergo dramatic changes in their morphometric and biological properties. Chondrocytes originate from condensed mesenchymal cells, which secrete matrixes rich in type II collagens and proteoglycan aggrecans, to form cartilage, which elongates via the proliferation and matrix production of its constituting chondrocytes. After coordinated and sequential differentiation, mature chondrocytes finally hypertrophy and undergo apoptotic cell death, and then, at that point, the cartilaginous mold is replaced by true bone by the osteoclasts and osteoblasts that come via the invading blood vessels.

Before the cartilaginous area is replaced by calcified bone, it is called the growth plate. The growth plate plays a crucial role in longitudinal-endochondral bone growth. Within the growth plate, sequential changes in cell size, shape, and orientation define distinct zones, i.e., resting, proliferative, and hypertrophic zones^3^. The resting zone consists of spherical cells that are randomly arranged and are considered to be the stem cells for the proliferative chondrocytes. The proliferative zone is composed of the proliferative chondrocytes flattened and aligned into columns, and these cells proliferate and synthesize extracellular matrices. Finally, the hypertrophic zone consists of hypertrophic chondrocytes with spherical shapes. These cells decrease the density of their cytoplasms and increase the volumes, which is followed by apoptotic cell death. The transient zone between the proliferative and hypertrophic zones is called the prehypertrophic zone.

The dynamic movement of chondrocytes in the growth plate has been observed by static histological analysis, which has two main limitations for investigating tissue growth and morphometric changes: one is that the fixing process of the tissues could alter the sizes of the cells and the extracellular matrices, and the other is that dynamic cellular movement can only be assumed from static histology of the different samples. Recently, a three-dimensional time-lapse movie system using two-photon excitation microscopy has enabled us to obtain dynamic imaging of a living organ and to analyze individual cell behavior^4^. In the present study, we adopted fetal murine ulnar explants to this live-imaging system and investigated the behavior and morphometric changes of chondrocytes in each zone of the growth plate. Further, as a positive intervention, we examined the effect of C-type natriuretic peptide (CNP), a known potent stimulator of endochondral bone growth on this system^5,6^, in order to extract the factors which contribute to stimulating the longitudinal growth of the growth plate cartilage.

## Results

### Dynamic growth plate imaging using cultured fetal murine ulna

**Figure 1:**
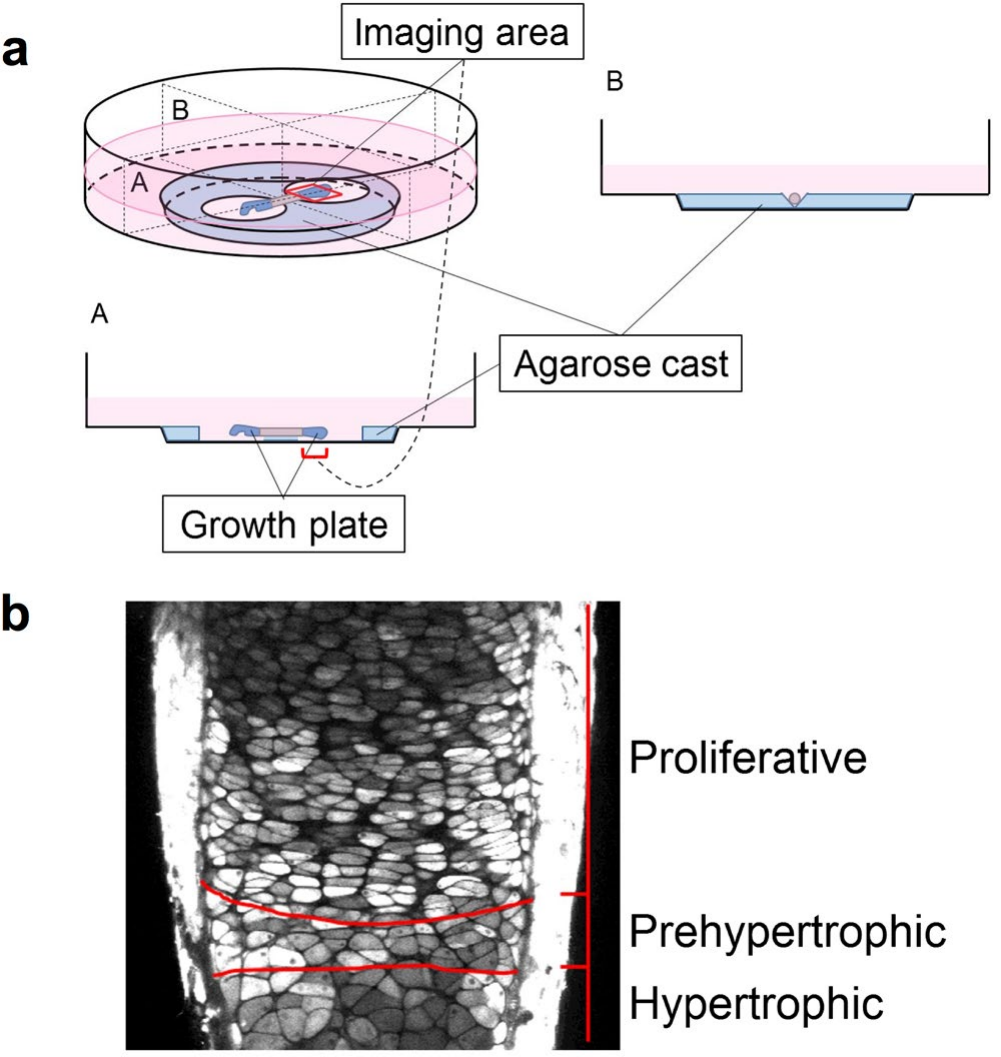
The dynamic imaging system of a fetal murine ulnar culture. (**a**) Setting of fetal murine ulnar culture. An explanted ulna was set on the agarose cast in the bottom of the glass-based dish, as shown in the schema. A red rectangle set on the distal growth plate, as indicated in the schema, represents an imaging area. The imaginary vertical-plane including longitudinal section of explanted ulna (A) and its maximal perpendicular vertical-plane (B) are indicated. (**b**) Definition of distinct areas of the growth plate in this imaging system. A captured photograph of the distal growth plate of explanted fetal murine ulna using two-photon excitation microscopy is shown. Red lines divide the growth plate into proliferative, prehypertrophic, and hypertrophic zones, as indicated.

To observe the time-dependent growth of the growth plate of mammalian long bone, we established a dynamic imaging system of a fetal murine ulnar explanted culture using two-photon excitation microscopy. The ulnar explant was dissected from a fetal mouse on gestational day 17 and set on the agarose cast in the bottom of a glass-based dish and incubated in culture medium. The explanted ulna was held and fixed at its calcified shaft and the freely-moving growth of distal cartilaginous primordium, i.e., growth plate cartilage, was steadily observed for 18 hours (Fig. 1a). From the beginning of the culture period, distinct areas including proliferative, prehypertrophic, and hypertrophic zones were clearly distinguished by the morphological and structural characteristics of the respective constituent chondrocytes (Fig. 1b). Time-lapse imaging of the explanted growth plate exhibited linear growth along the long bone axis during the 18-hour experimental period (see Supplementary Video). The whole length of the distal ulnar growth plate was increased by 179.4 ± 9.5 μm (n = 5) during the experimental period. We histologically confirmed that the extension and cell morphology of growth plates that were exposed or unexposed by fluorescence did not show obvious differences, indicating that photo-toxicity was negligible in this experiment (see Supplementary Figure for representative histological pictures). Supplementation of CNP at a dose of 10^−7^ M to the culture in our imaging system stimulated the growth of the ulnar growth plate cartilage (see Supplementary Video). The whole length of the distal ulnar growth plate in the CNP-treated group was increased by 249.5 ± 10.9 μm (n = 5) and was significantly larger than that of the non-treated group (p < 0.01), as was consistent with previous reports^5,6^.

As is shown in Fig. 1b, the prehypertrophic zone is quite thin and the chondrocytes there might promptly undergo hypertrophic change. Therefore, we designated the zone as “prehypertrophic-hypertrophic” in the experiments with the longer observational period (18 hours). On the other hand, we mentioned it only “prehypertrophic” in those with the shorter observational period (6 hours), regarding the chondrocytes there are retained in the same zone and remain relatively unchanged after a short period like 6 hours.

### Cell displacement length analyses of chondrocytes in each zone of the growth plate

**Figure 2:**
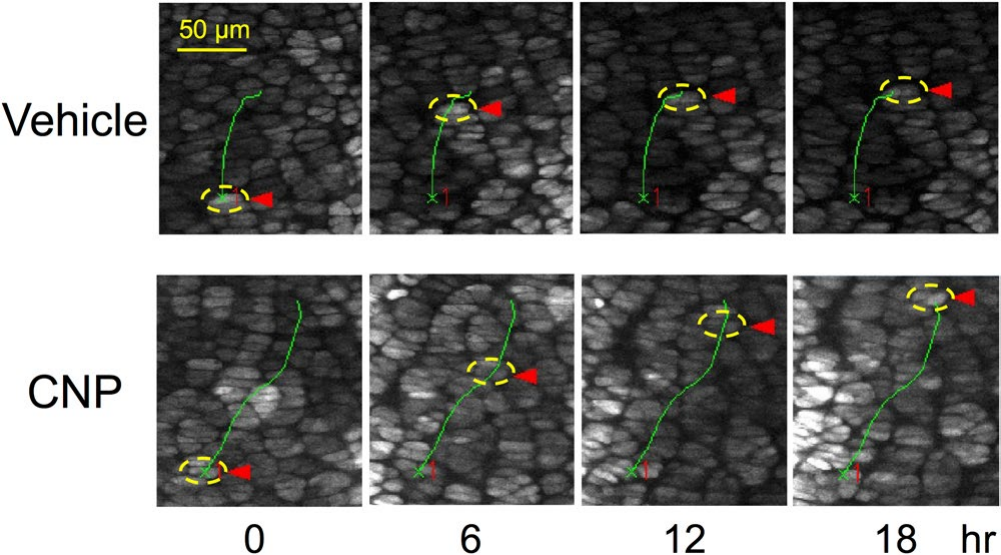
Trajectory of a chondrocyte in the proliferative zone in vehicle (upper panels) or CNP (lower panels)-treated growth plate organ culture. Pictures in each vertical lane represent the snap shots at indicated time point (hour). Targeted chondrocyte is encircled by yellow dashed ellipse marked by red arrowhead at each time point, which is located on the full trajectory indicated by the same green line, appearing in each set of pictures. The ‘x’ numbered ‘1’ in red character in each snap shot exhibits the start point of the trajectory of the targeted chondrocyte in vehicle or CNP-treated group.

We tried to analyze the factors which contributed to the elongation of the growth plate cartilage observed in this live-imaging system. As a major component of the linear growth of the growth plate cartilage, we measured the displacement of each cell in the growth plate. A representative trajectory of a single chondrocyte in the proliferative zone is shown in Fig. 2 and the Supplementary Video.

**Figure 3:**
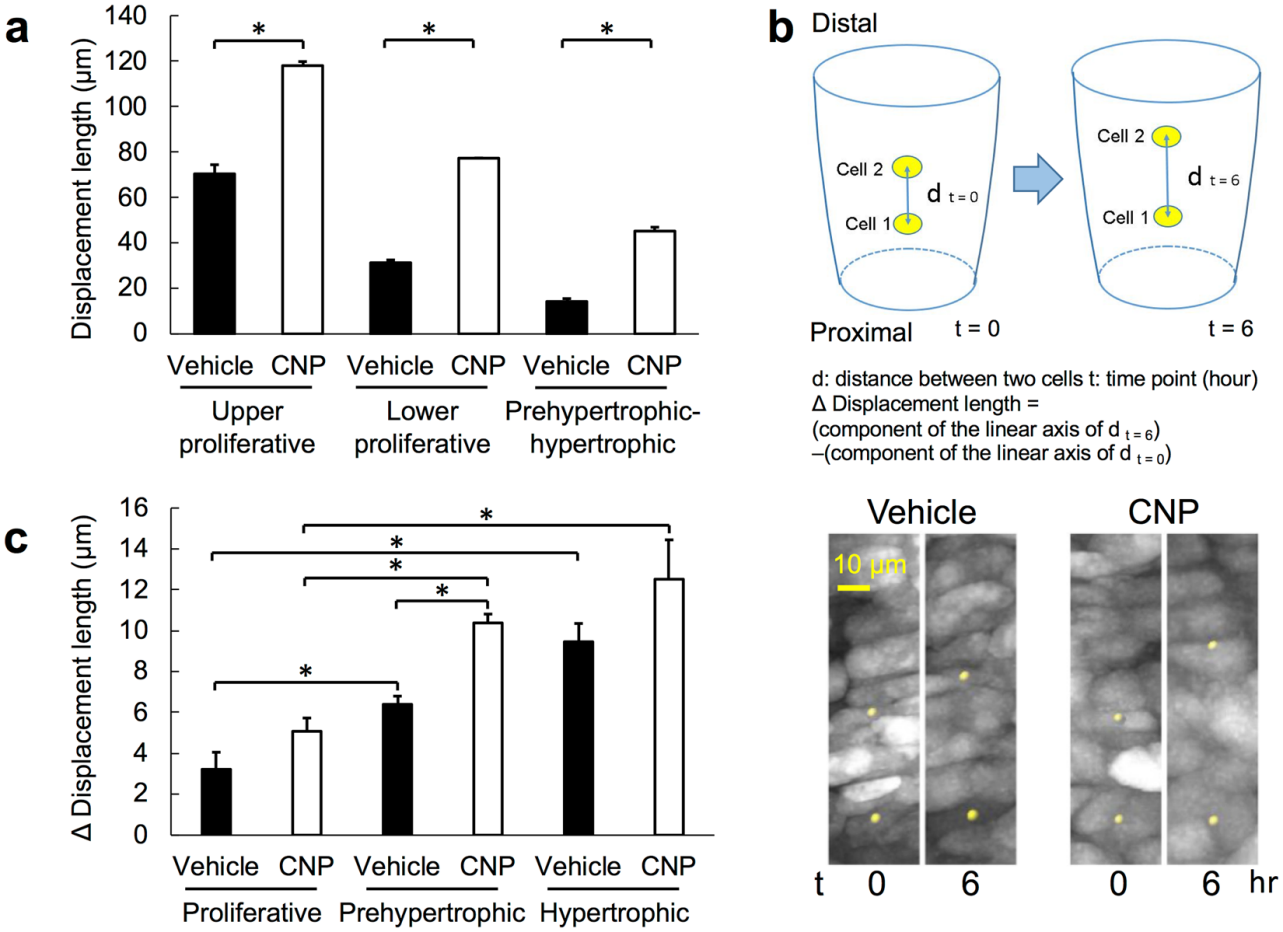
Cell displacement length analyses, based on a Cartesian coordinate system (see “Methods”). (**a**) The displacement length of each chondrocyte measured as the distance along the longitudinal growth of the growth plate, set the boundary line between the calcified bone shaft and the growth plate cartilage as the baseline. Results of chondrocytes in distinct zones of the growth plate are shown. (**b**) Upper panel: a schema representing the displacement length of chondrocytes defined as the distance between two distinct chondrocytes. Each outer frame represents the distal ulnar growth plate and the yellow ellipse represents the chondrocyte. d: distance between two cells, t: time point (hour), Δ displacement length = (component of the linear axis of d t = 6)−(component of the linear axis of d t = 0). Lower panels: representative views of marked chondrocytes (yellow points) at t = 0 and t = 6. Left: vehicle-treated and right: CNP-treated. (**c**) Δ displacement length of two distinct chondrocytes in the proliferative, prehypertrophic, and hypertrophic zones. Closed and open bars represent vehicle and CNP-treated groups, respectively. (**a**) and (**c**), n = 5, *P < 0.05.

At first, as we fixed the calcified bone shaft of the explanted ulna in this organ culture system, we set the boundary line between the calcified bone shaft and the growth plate cartilage as the baseline of measurement. We then estimated the distance of displacement of a single chondrocyte in each zone (upper and lower proliferative, and prehypertrophic-hypertrophic) as the difference between components of the bone growth axis at time point 0 and 18 hours (see Methods). Understandably, the displacement lengths by this definition increased proportionally to the initial distances of the concerned chondrocytes from the baseline; these values were the largest in the upper proliferative chondrocytes and the least in prehypertrophic-hypertrophic chondrocytes (Fig. 3a). Furthermore, CNP significantly increased the displacement length of the chondrocytes in each zone of the growth plate (Fig. 3a).

Next, we evaluated the displacement of the chondrocytes based on the distance between two distinct chondrocytes in the same growth plate zone (Fig. 3b,c). As represented in Fig. 3b, the distance between two chondrocytes was measured, and the difference of the component of the axis along linear bone growth during the 6-hour incubation time was defined as delta (Δ) displacement length. The Δ displacement length of chondrocytes in the prehypertrophic or hypertrophic zone was significantly larger than that in the proliferative zone (Fig. 3c). In this setting, CNP tended to but did not significantly increase the Δ displacement lengths in the proliferative and hypertrophic zones, and significantly increased the length in the prehypertrophic zone (Fig. 3c).

### Analysis of the alteration of segmented areas in proliferative and prehypertrophic-hypertrophic zones

**Figure 4:**
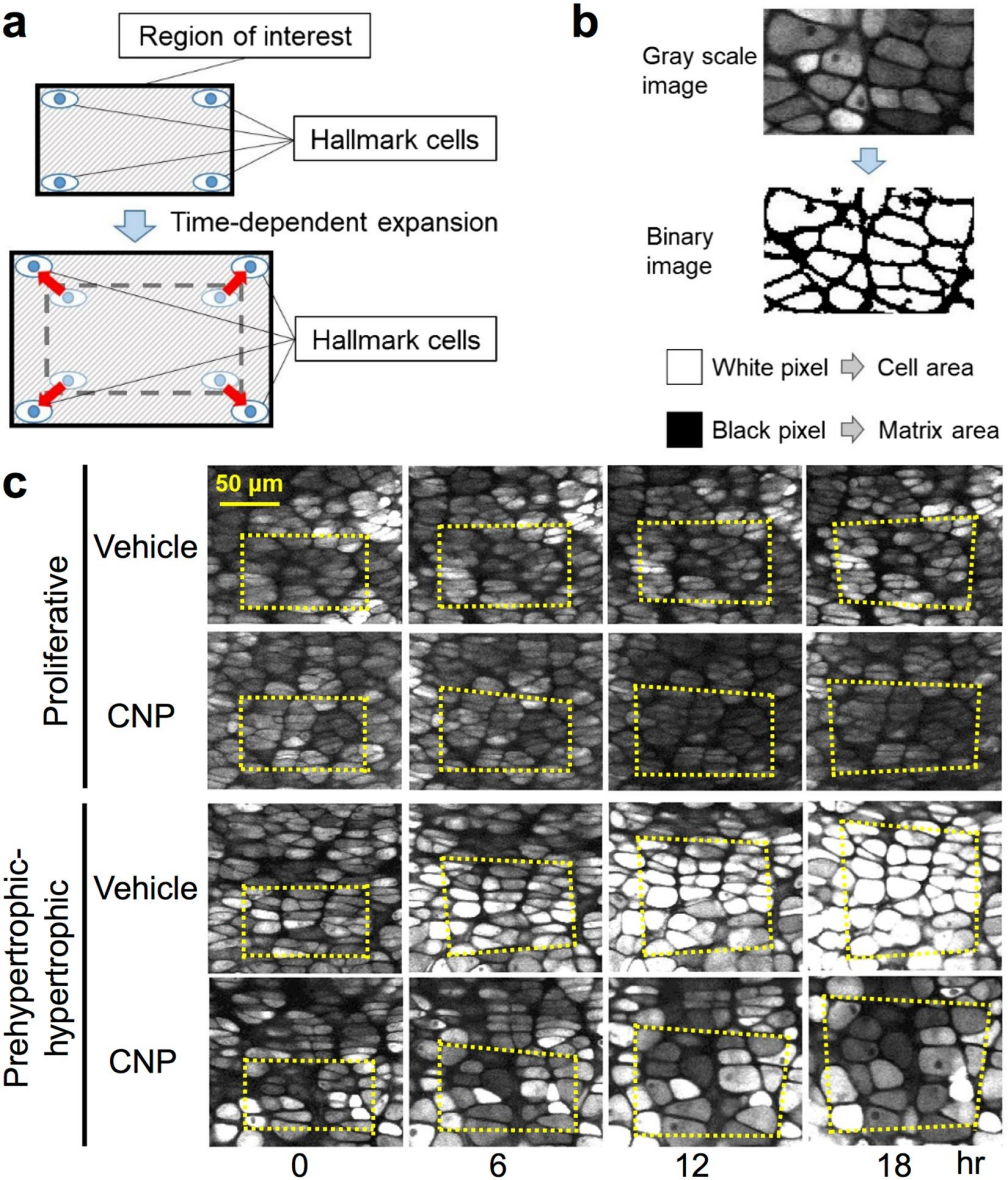
Schematic representation of the methods of pixel analyses (**a**,**b**) and the practical images used in the analyses (**c**). (**a**) Hallmark cells were set in the corner of the region of interest (ROI) at time point 0 hours; an area of ROI at time point 0 hours was set about 6,000 pixels. The ROI at each time point was cropped based on the hallmark cells. (**b**) Binarization processing using MetaMorph. Cell areas were set as white pixels and extracellular matrixes as black pixels. (**c**) Practical images used in pixel analyses in the proliferative (upper panels) and prehypertrophic-hypertrophic (lower panels) zones. In series of pictures in each zone, vehicle-treated (upper pictures) and CNP-treated (lower pictures) are indicated at each time point. Yellow dashed quadrangle indicate the ROIs.

**Figure 5:**
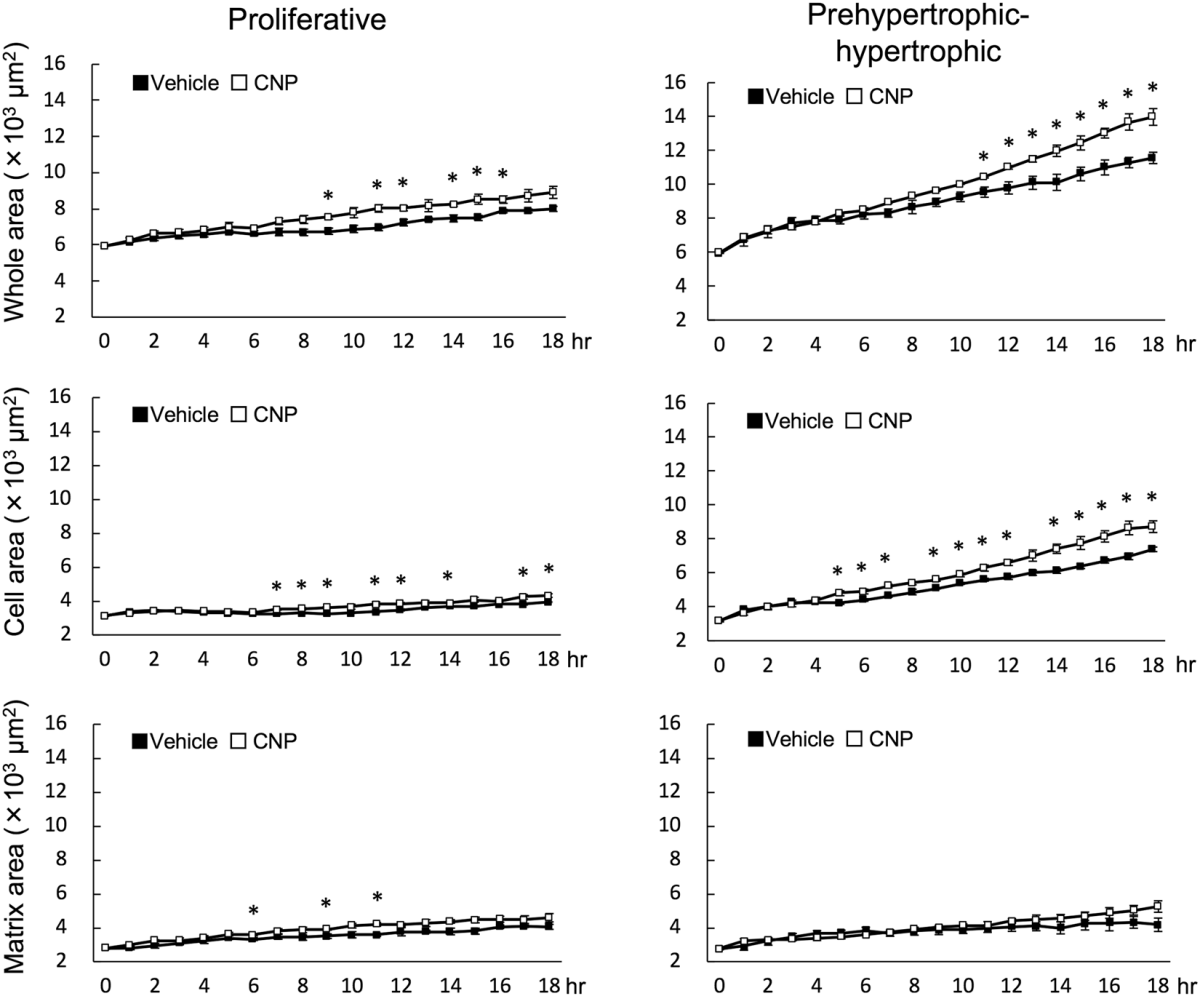
Pixel analyses of whole and segmented areas in the growth plate. Left panels represent the results of the proliferative zones and right panels show those of the prehypertrophic-hypertrophic zones. Upper, middle, and lower panels show the whole area, segmented cellular, and matrix areas, respectively. Pixel analyses were performed using binary images for 18 hours. Closed and open circles represent vehicle and CNP-treated groups, respectively. n = 3, *P < 0.05.

Then, as a contributing factor to elongation of the growth plate cartilage, we evaluated the time-course transition of cellular and extracellular components in a segmented area in the proliferative or prehypertrophic-hypertrophic zones during the 18-hour experimental period (the method and the practical images are shown in Fig. 4). At the start of the experiment (time point 0-hour), a segmented area was set about 6,000 pixels (about 6,000 μm^2^), and the ratios of the cellular area to the whole segmented area were similar between in proliferative and prehypertrophic-hypertrophic zones (52.9 ± 0.6% and 53.6 ± 1.0%, respectively, n = 6, each). Whole segmented areas in both the proliferative and prehypertrophic-hypertrophic zones were gradually increased, along with their respective extracellular matrix areas (Fig. 5, upper and lower panels). The increase in the cellular area in the proliferative zone was less obvious than that in the prehypertrophic-hypertrophic zone (Fig. 5, middle panels).

The addition of CNP fundamentally increased the whole segmented areas in the growth plate; at the end of the 18-hour experimental period, CNP had significantly increased the whole segmented area in the prehypertrophic-hypertrophic zone, whereas the increase was not significant in the proliferative zone (Fig. 5, upper panels). Furthermore, at most time points in the latter half of the experimental period, cellular areas were significantly increased by CNP in both the proliferative and the prehypertrophic-hypertrophic chondrocyte layers (Fig. 5, middle panels). At that time, the extracellular areas in both the proliferative and prehypertrophic-hypertrophic zones of the CNP-treated group were not significantly greater than those of the vehicle-treated group at most time points, although an increasing tendency was observed (Fig. 5, lower panels).

### Changes in single cell areas in proliferative, prehypertrophic, and hypertrophic zones

**Figure 6:**
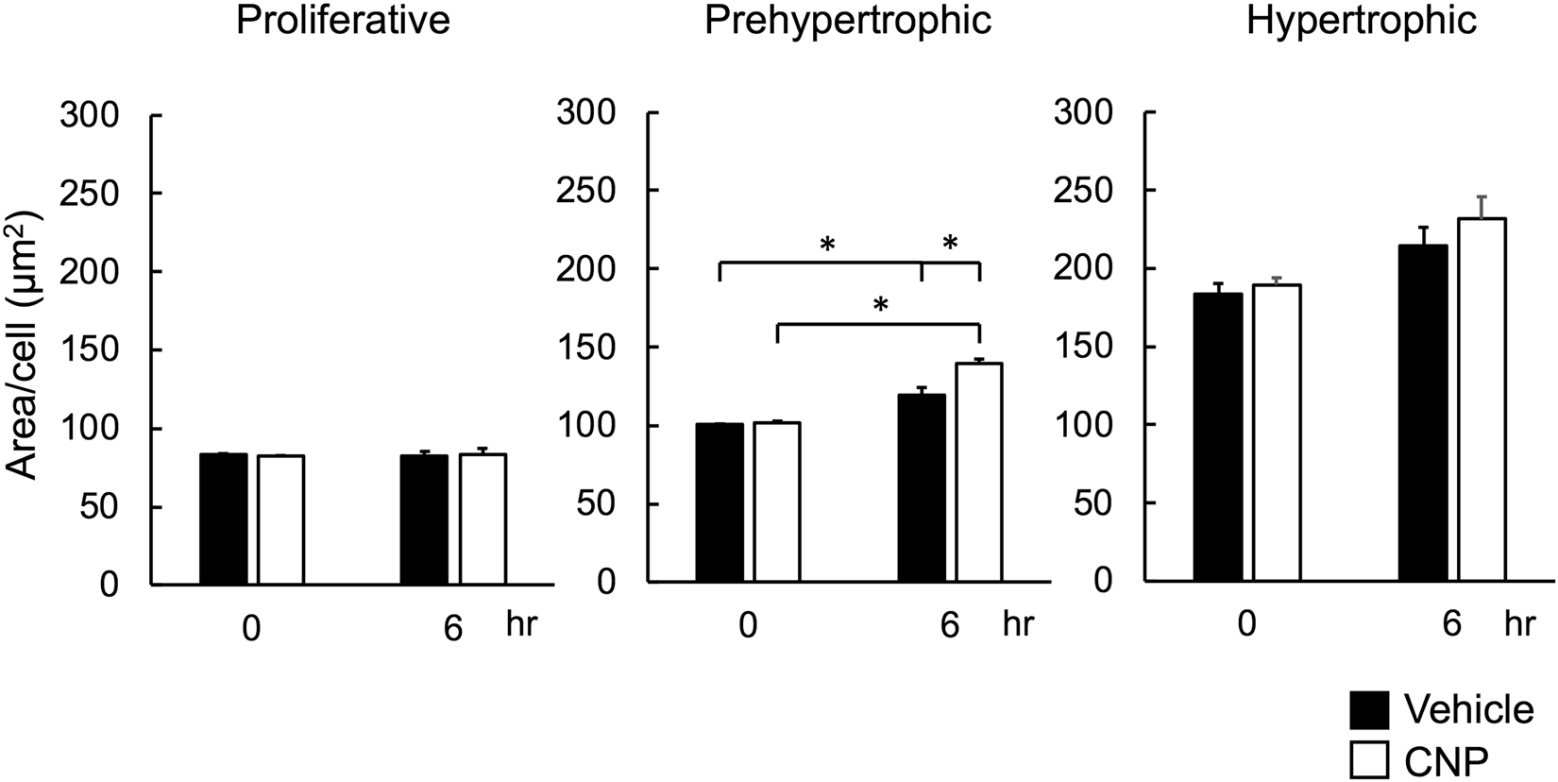
Pixel analyses of single cell areas. Graphs of the average areas of each chondrocyte in the proliferative (left), prehypertrophic (middle), and hypertrophic (right) zones. Analyses were performed at the start of the experiment and at 6 hours. Closed and open bars represent vehicle and CNP-treated groups, respectively. n = 3, *P < 0.05.

Further, we analyzed the alteration of the area of a single chondrocyte in the proliferative, prehypertrophic, and hypertrophic zones. A single cell area was represented as the quotient of the total cell area divided by the cell number in a segmented area, and the values at time points 0 and 6 hours were evaluated. At time point 0 hours, the single cell area was 82.1 ± 0.5, 100.4 ± 0.7 and 185.4 ± 3.7 μm^2^ in proliferative, prehypertrophic, and hypertrophic zones, respectively (n = 6, each) (Fig. 6). In accordance with the results of the analysis of the segmented areas in the growth plate, the single cell area in the proliferative zone did not change after the 6-hour incubation period (Fig. 6, left panel). On the other hand, the single cell area in the prehypertrophic zone was significantly increased after the 6-hour incubation period (Fig. 6, middle panel). In the hypertrophic zone, the single cell area tended to be increased after the 6-hour incubation period, but the difference was not significant (Fig. 6, right panel). The single cell area in the proliferative zone did not change, whereas that in the prehypertrophic zone was significantly increased, by supplementation of CNP for 6 hours (Fig. 6, left and middle panels). CNP tended to but did not significantly increase the single cell area in the hypertrophic zone (Fig. 6, right panel).

### Cell division rates of proliferative chondrocytes

**Figure 7:**
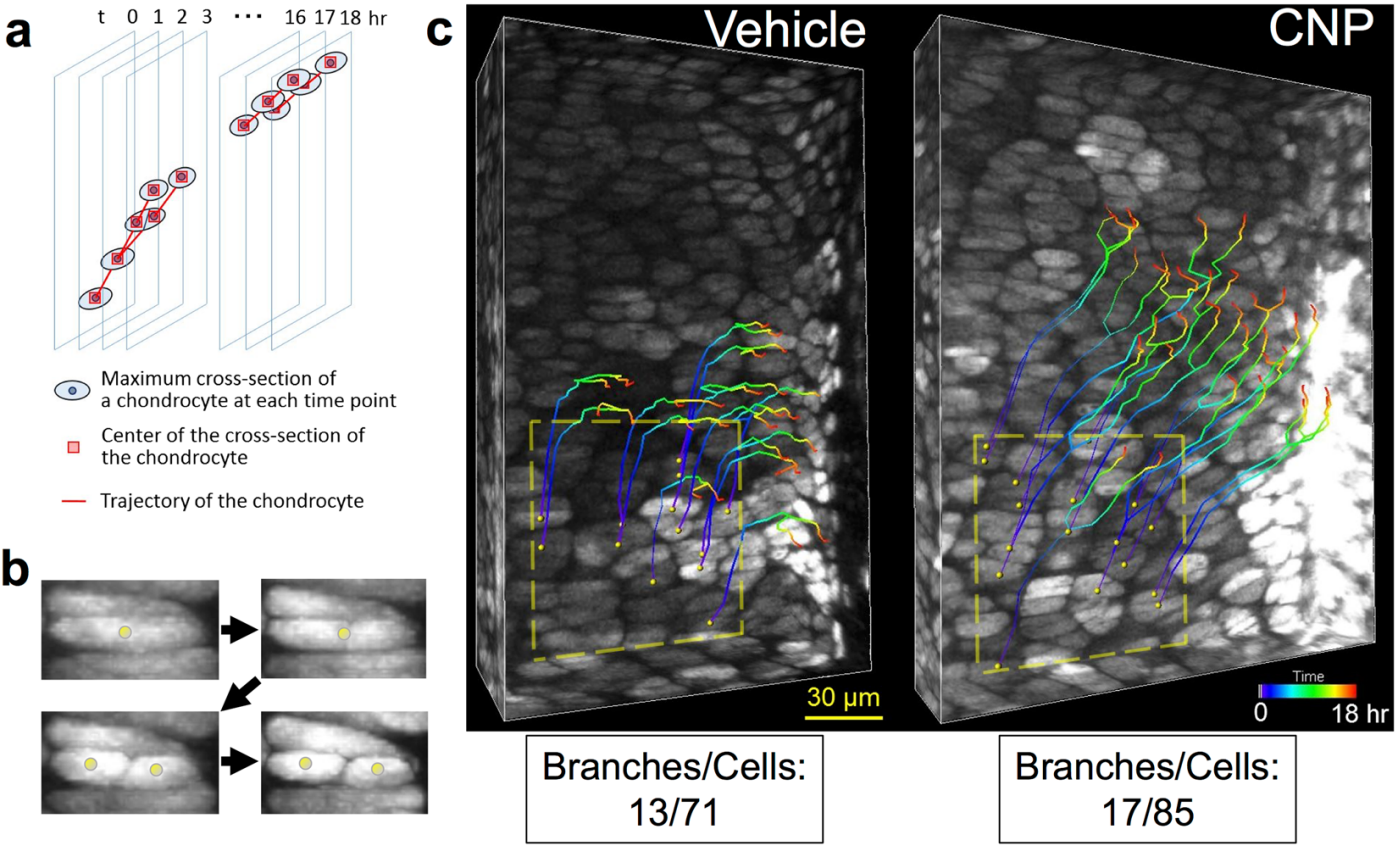
Analysis of the proliferation of growth plate chondrocytes using IMARIS. (**a**) Schema of the method. The maximal cross-section of a targeted chondrocyte was provided at each time point, and the center of the cross-section of the chondrocyte was manually connected for 18 hours using IMARIS. Mitotic division is represented as a branched line, as indicated. (**b**) A representative series of pictures showing the division of a proliferative chondrocyte. Yellow spheres indicate the centers of the maximal cross-sections of chondrocytes. (**c**) All proliferative chondrocytes located within a distinct rectangle (depicted by yellow dashed lines) in an imaginary plane were traced, but only the trajectories of dividing chondrocytes are shown. Left, vehicle and right, CNP-treated groups. Colored lines represent trajectories of proliferating chondrocytes divided during the 18-hour observation period. Each branch of the line means mitotic division of a proliferative chondrocyte. The total numbers of branches by all cells traced in vehicle and CNP-treated groups are also indicated.

The proliferation of chondrocytes could be a contributor to the elongation of the growth plate cartilage. Therefore, we estimated the proliferation of chondrocytes in the proliferative zone by analyzing the division rate of proliferative chondrocytes in time-dependent 4D images using the software IMARIS. A 100 μm square area was constructed in the proliferative zone in a 4D image, and each chondrocyte there was traced during the 18-hour experimental period (the schematic representation of the method and the practical image of a dividing cell are depicted in Fig. 7a,b, respectively). The overall number of cells and the total number of branches included in the area of analysis were 71 and 13, respectively, thus the cell division rate was 18.3% (Fig. 7c, left panel). In the case of CNP treatment, the overall number of cells and total number of branches included in the area of analysis were 85 and 17, respectively (Fig. 7c, right panel). Therefore, the cell division rate in the CNP-treated group was 20.0%, which was similar to the value in the non-treated group.

## Discussion

The growth plate is crucial for long bone growth and the observation of its dynamic growth is of high interest. In 2014, Romereim *et al*. investigated the dynamic behavior of a living growth plate at the murine presphenoidal synchondrosis using time-lapse confocal microscopy^7^. As for the study on the dynamic imaging of the growth plate in long bones, Li *et al*. reported the live-imaging analysis of the growth plate of avian embryonic metacarpal bones in a tissue culture system using time-lapse two-photon laser scanning microscopy^8^. In the present study, we have established a live-imaging system for investigating the dynamic growth of the growth plate of mammalian long bones in a fetal murine ulnar culture using the same time-lapse two-photon laser scanning microscopy which Li *et al*. had adopted; this is the first report on the analysis of dynamic growth of the living growth plate in mammalian long bones.

As for the factors contributing to the elongation of the growth plate, we first estimated the displacement length of each chondrocyte. At first, as we fixed the calcified shaft of the ulnar explant in our live-imaging system, we set the edge of the growth plate cartilage (the line between calcified bone shaft and growth plate cartilage) as the baseline of the measurement, assuming that the calcified bone part of an ulnar explant would not move nor elongate. In this setting, the displacement of a chondrocyte, estimated as the component of coordinate length along the axis of linear growth of the cultured ulna, becomes the sum of the movement of the component (cells and extracellular matrices) between the intended cell and the baseline. Therefore, it is a matter of course that the displacement length became larger in accordance with the distance between the initial location of the objective chondrocyte and the baseline. In addition, the baseline could move and be unstable for technical reasons in spite of our careful attention. Because of these reasons, in addition to measuring the displacement from baseline, we estimated the change of two-cell distance as the Δ displacement length; the Δ displacement length was significantly larger in the prehypertrophic or hypertrophic zone than in the proliferative zone, suggesting a greater contribution of prehypertrophic or hypertrophic chondrocytes than proliferative chondrocytes for growth plate elongation.

Secondly, we evaluated the cellular and extracellular spaces in the proliferative and prehypertrophic-hypertrophic zones for the causative factor for the above-mentioned displacement of each chondrocyte. The increase in the cellular area, as well as in the whole preset area, was greater in the prehypertrophic-hypertrophic than in the proliferative zone, which would owe to the larger Δ displacement length of the chondrocyte in the prehypertrophic or hypertrophic zone than in the proliferative zone. The result of the single cell analysis further supports this notion, i.e., the single cell area was significantly and tended to be increased in the prehypertrophic and hypertrophic zones, respectively, whereas it was not changed in the proliferative zone, after the 6-hour incubation period.

One of the most important points of our present study is that we used CNP as a known positive stimulator of growth plate elongation. We and another group had previously reported the potent stimulating effect of CNP on the growth of cartilaginous primordia, i.e., growth plate cartilage, in *ex vivo* tissue culture systems using fetal murine long bones^5,6^. Noteworthy, the bioactive receptor for CNP, natriuretic receptor-B (NPR-B), is expressed in both proliferative and prehypertrophic zones, and more strongly expressed in prehypertrophic than in proliferative zone^9^. Our present observation showed that the CNP-induced stimulation of the elongation of growth plate cartilage is due to the increases in the cellular and also somewhat in the matrix areas in the proliferative, and more potently in the prehypertrophic-hypertrophic zones, which might coincide with the fact that NPR-B is more strongly expressed in prehypertrophic zone than in proliferative zone. On the other hand, CNP did not have a potent effect on the proliferation of chondrocytes in the proliferative zone, as shown by the result of IMARIS in this study, which coincides with the results of our previous reports describing that CNP does not have a strong effect on the stimulation of chondrocyte proliferation^10,11^. Previously, Li *et al*. concluded in their ingenious report that increased extracellular matrix deposition and cellular volume enlargement are the key contributors to cartilage elongation^8^. The manner in which CNP elongates growth plate cartilage agrees nicely with their conclusion, and CNP may be an authentic stimulator of endochondral bone growth in accordance with the theory advocated by Li *et al*.

As for the weakness of our present study, because cell size and shape constantly change in the growth plate as a dynamic structure, it might be difficult to generalize the results from small number of cells, especially over a relatively short period of time as reported here, and fully figure out the mechanistic insight into the elongation of growth plate or the endochondral bone growth. Furthermore, the weaknesses of our present study includes the estimation of chondrocyte proliferation by IMARIS, in that analysis was performed using a single set of cells in the vehicle or CNP-treated group. To perform manual tracking for all chondrocytes and estimate their division in our defined area was so hard that it was technically difficult to carry out this task in multiple samples. In order to elicit a more statistically accurate conclusion on the effect of CNP on chondrocyte proliferation using IMARIS, we might have to set multiple samples with much smaller areas and perform the same analysis in each sample.

Although we established a live-imaging system of growth plate cartilage using murine explanted cartilaginous primordia, it is limited to observing only one process of endochondral bone growth; the other major processes include the replacement of cartilaginous primordia by calcified bone tissue, the apoptosis of hypertrophic chondrocytes, vascular invasion to the apoptotic areas, and the following migration of osteoblasts and osteoclasts through blood vessels. So, the next step of our live-imaging analysis for endochondral bone growth should aim for the observation of the whole process, maybe by using an *in vivo* animal model^12,13^. We are now preparing for it in our laboratory.

In conclusion, we established a live-imaging system for the observation of the elongation of growth plate cartilage in cultured-murine long bones using two-photon excitation microscopy, and analyzed the factors contributing to the elongation, in the setting with or without CNP. Utilizing other reagents in this culture system and the subsequent alteration of the behavior of each chondrocyte or whole growth plate might shed light on novel mechanisms of endochondral bone growth, which may lead to the discovery of a novel strategy for the treatment of patients suffering from disorders with impaired skeletal growth.

## Methods

### Organ culture and dynamic imaging of ulnar growth plates

All experimental procedures using mice were performed in accordance with the ethical guidelines of Kyoto University, and this study was approved by the Animal Research Committee, Graduate School of Medicine, Kyoto University (Med Kyo 17218). C57BL/6-Tg (CAG-EGFP) mice were purchased from Shimizu Experimental Supplies (Kyoto, Japan). The mice express EGFP in almost all body cells under the control of CAG promoter^14^. Between 10 a.m. to 12 a.m. on day 17 of pregnancy, mice were sacrificed by cervical dislocation. Bilateral ulnas were aseptically dissected from mouse fetuses. Ulnas were trimmed intact in order to not damage the perichondrium. Organ cultures of fetal mouse ulnas were performed by the static culture technique in a BGJb medium (Thermo Fisher Scientific, Massachusetts, USA) containing 6 mg/ml BSA (Wako Pure Chemical Industries, Osaka, Japan), 150 μg/ml ascorbic acid (Wako Pure Chemical Industries), 100 units/ml penicillin, and 100 μg/ml streptomycin (Wako Pure Chemical Industries). No serum was added to the medium. Ulnar explants were incubated in 35 mm glass-based dishes (Asahi glass, Tokyo, Japan). Ulnar explants were set on the agarose grooves cast in the bottom of the dishes, of which the region around both ends of the ulnas was removed so as to not inhibit their physiological growth. The metaphysis of the specimen was held by the agarose cast to avoid an artificial movement. Distal growth plates were observed using inverted two-photon laser excitation microscopy with a CO2 incubator (LCV110-MPE, Olympus, Tokyo, Japan). For imaging chondrocytes expressing EGFP, two-photon excitation was used with 16–23% relative powers at a wavelength of 930 nm. Image size was set at 512 by 512 pixels, with each pixel size at 0.994 by 0.994 μm, and thickness of each plane at 2 μm. Time-lapse imaging was performed for 6 hours (for Δ displacement length measurement and analysis of the single cell area) or 18 hours (for other experiments), with vehicle or CNP (10^−7^ M). Additionally, to validate the elongation of the growth plate cartilage with vehicle or CNP, whole lengths of distal ulnar growth plates were measured at time points 0 and 18 hours using a linear ocular scale mounted on a dissecting microscope.

### Histology and definition of proliferative, prehypertrophic, and hypertrophic zones

Fetal mouse ulnas were fixed in 10% formaldehyde neutral buffer solution (Nacalai tesque, Kyoto, Japan) for 24 hours and decalcified by G-Chelate Mild (Genostaff, Tokyo, Japan), then embedded in paraffin. 4 μm thick sections were cut from paraffin-embedded specimens, and stained with hematoxylin/eosin. Each segmented area of the growth plate was defined by comparison between histology acquired from static and dynamic analysis. Areas composed of enlarged cytoplasm were defined as hypertrophic zones. Areas adjoining to hypertrophic zones and without enlargement of cytoplasm or columnar array structures were defined as prehypertrophic zones. Further, areas adjacent to prehypertrophic zones and composed of flattened and columnar arrayed cells were defined as proliferative zones. The height of the proliferative zone was measured, and the proliferative zone was divided in half to produce upper and lower proliferative zones^3^.

### Measurement of cell displacement length

To analyze cell displacement, we fundamentally supposed a Cartesian coordinate system as described in ref.^8^: we defined a proximo-distal axis of the ulna as the y-axis, the axis perpendicular to the y-axis in the imaging plane as the x-axis, and the axis orthogonal to the imaging plane as the z-axis. Then, we estimated cell displacement length in the following two ways: (1) Assuming that the calcified bone shaft is stable and does not move, we set the boundary line between the calcified bone shaft and the growth plate cartilage (i.e., border line at the end of the hypertrophic zone) as 0 on the y-axis and the baseline of the measurement. Following this, we defined the displacement length of a chondrocyte as the difference between the y-axis measurements at the start and end of the experimental period. We analyzed five chondrocytes in the same imaging plane perpendicular to the y-axis in the proliferative or prehypertrophic-hypertrophic zone in vehicle or CNP-treated specimen. (2) At the start of analyses, five pairs of chondrocytes, of which the distance between each pair of chondrocytes measured along the y-axis was around 30 μm and the proximal one is located on the same imaging plane perpendicular to the y-axis, were selected in the proliferative, prehypertrophic, and hypertrophic zones. Each pair of cells was tracked over time using IMARIS (Carl Zeiss, Jena, Germany), then the differences in the distances of each pair of cells at time points 0 and 6 hours were calculated. In both (1) and (2), analyses were performed on three specimens in the vehicle or CNP-treated groups.

### Pixel analysis

The time-lapse images were imported as Tiff files to MetaMorph (Molecular Devices, California, USA). To analyze time-dependent changes of cell and matrix areas, hallmark cells were set in the corner of the region of interest (ROI) at time point 0 hours. ROI was set as a rectangle of which area included 6,000 (100 W × 60 H) pixels, then images at each time point were cropped based on the hallmark cells (Fig. 4a). Cropped images were binarized using Adaptive Threshold, which is a built-in program of MetaMorph. The same threshold was adopted to convert gray scale images into binary images throughout the experiment. We set the cell areas as white pixels and the extracellular matrixes as black pixels, and counted them at each time point (Fig. 4b). The analyses were performed in the proliferative and prehypertrophic-hypertrophic zones, either treated with or without CNP.

To assess the change in volume of each cell, we measured single cell areas. ROI was set as a rectangle of which area included 12,000 (200 W × 60 H) pixels in proliferative and prehypertrophic zones and 6,000 (100 W × 60 H) pixels in hypertrophic zone. Hallmark cells were set in the corner of ROI at time point 0 hours, then traced to time point 6 hours. Cell number in the ROI was counted in gray scale images by visual inspection. The total cell area in the ROI was counted in binary images, then the single cell area was calculated as a quotient of total cell area by cell number. Analyses were performed in three different growth plate samples of the proliferative, prehypertrophic, and hypertrophic zones, with or without CNP treatment.

### Measurement of mitotic divisions of proliferative chondrocytes

Mitotic divisions of proliferative chondrocytes were counted in 4D images using IMARIS. Representative areas in the proliferative zones were extracted for 100 μm squares, then the chondrocytes included in these areas were tracked through the experiments. The total numbers of tracks and branches were counted and cell division rates were calculated in the vehicle and CNP-treated groups.

### Statistics

Data were expressed as the mean ± S.E. The statistical significance of differences in mean values was assessed by Student’s t test or two-way factorial analysis of variance (ANOVA), followed by Tukey-Kramer test as a post hoc test. Differences among means were considered significant at values of p < 0.05 or 0.01.

### Data availability

All data generated or analyzed during this study are included in this published article and its Supplementary Information files.

## Electronic supplementary material

Supplementary Information

Supplementary Video
