# Supplementary material for: Live imaging analysis of the growth plate in a murine long bone explanted culture system
Source: Sci Rep. 2018 Jul 9;8:10332. doi: 10.1038/s41598-018-28742-x (PMC6037772; doi:10.1038/s41598-018-28742-x)

Supplementary Information

Live imaging analysis of the growth plate in a murine long bone explanted culture system

Keisho Hirota, Akihiro Yasoda, Yugo Kanai, Yohei Ueda, Ichiro Yamauchi, Takafumi Yamashita, Yoriko Sakane, Toshihito Fujii and Nobuya Inagaki

Supplementary Figure: Histological Picture of distal growth plates in fetal murine ulnar explants after 18-hour culture period. Growth plates unexposed (upper picture) or exposed (lower picture) by fluorescence are shown.

Supplementary Video: Time-lapse imaging of the explanted fetal murine ulnar growth plates for 18 hours. Red arrowheads indicate representative chondrocytes in the proliferative zone (proliferative chondrocytes), and their trajectories are shown in green lines. Vehicle-treated (left) and CNP-treated (right) explants are shown.

Supplementary Figure

Control

LCV-MPE

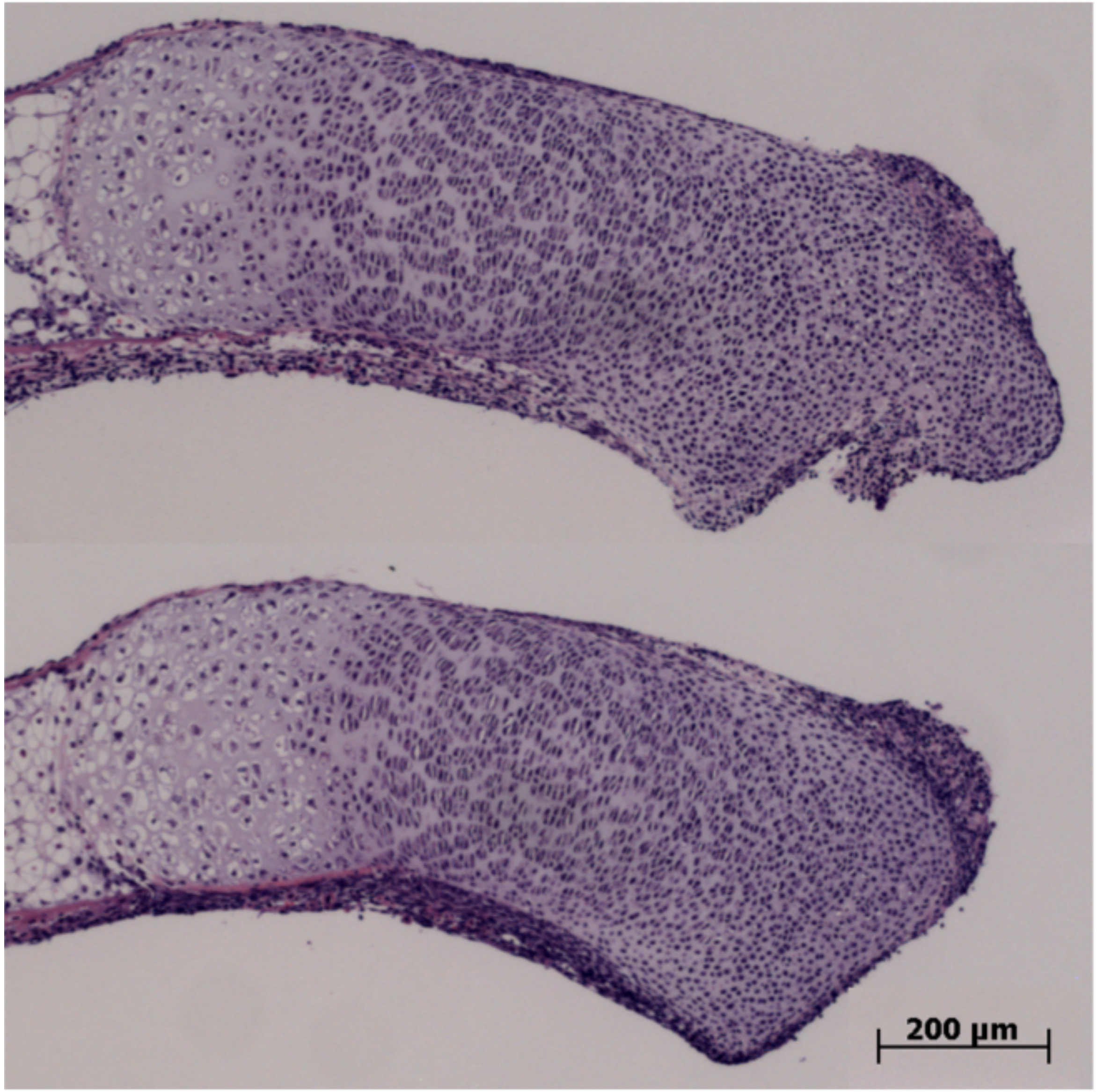

Supplement: Supplementary file 1 — Supplementary Information [file 41598_2018_28742_MOESM1_ESM.pdf]
